# Correction

**DOI:** 10.1080/14756366.2023.2255447

**Published:** 2023-09-04

**Article title**: Discovery of novel arylamide derivatives containing piperazine moiety as inhibitors of tubulin polymerization with potent liver cancer inhibitory activity

**Authors:** Xiao-Yi Shi, Huang Jiao, Jia-Kai Zhang, Xin-Yi Tian, Dan-Feng Guo, Jie Gao, Mei-Qi Jia, Jian Song, Sai-Yang Zhang, Xiang-Jing Fu, Hong-Wei Tang

**Journal:** *Journal of Enzyme Inhibition and Medicinal Chemistry*

**Bibliometrics:** Volume 38, Number 1,

**DOI:** https://doi.org/10.1080/14756366.2023.2237701

The authors regret the following errors in **Figure 5A ∼ C** of this article. The authors would like to apologize for any inconvenience caused. The correct version of **Figure 5** is as follows:

**Figure 5.**
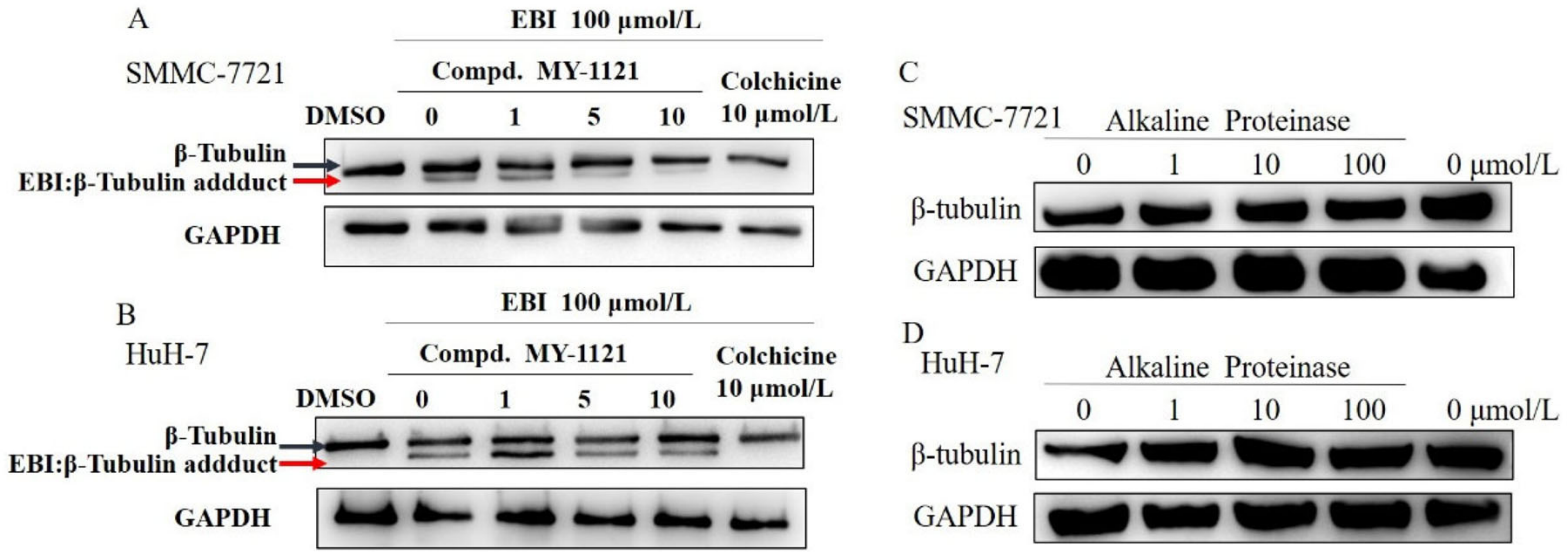
Compound **MY-1121** bind to β-tubulin directly on colchicine binding site. (A&B). EBI competition assay, the affinity of the compound with colchicine binding site was negatively correlated with the level of the tubulin adduct band; (C&D). Alkaline protease assay. Cells were treated with Alkaline Proteinase and different concentration of compound **MY-1121**. The band signal of β-tubulin was negatively correlated with the ability of Alkaline Protease to hydrolyze β-tubulin. The binding of the compound to β-tubulin was able to inhibit the hydrolysis of β-tubulin by Alkaline Protease.

